# Addressing health disparities in Hispanic breast cancer: accurate and inexpensive sequencing of *BRCA1* and *BRCA2*

**DOI:** 10.1186/s13742-015-0088-z

**Published:** 2015-11-04

**Authors:** Michael Dean, Joseph Boland, Meredith Yeager, Kate M. Im, Lisa Garland, Maria Rodriguez-Herrera, Mylen Perez, Jason Mitchell, David Roberson, Kristine Jones, Hyo Jung Lee, Rebecca Eggebeen, Julie Sawitzke, Sara Bass, Xijun Zhang, Vivian Robles, Celia Hollis, Claudia Barajas, Edna Rath, Candy Arentz, Jose A. Figueroa, Diane D. Nguyen, Zeina Nahleh

**Affiliations:** 1 Laboratory of Experimental Immunology, National Cancer Institute, Frederick, MD 21702 USA; 2 Cancer Genetics Research Laboratory, Division of Cancer Epidemiology and Genetics, National Cancer Institute, Gaithersburg, MD USA; 3 Basic Science Program, Leidos Biomedical Research, Inc., Frederick, MD USA; 4 Nueva Vida Richmond, Richmond, VA USA; 5 Latino Community Development Agency, Oklahoma City, OK USA; 6 Texas Tech University Health Sciences Center, El Paso, TX USA; 7 Texas Tech University Health Sciences Center, Lubbock, TX USA

**Keywords:** Breast cancer, Hispanic populations, Genetic testing, Underserved populations, Health disparity

## Abstract

**Background:**

Germline mutations in the *BRCA1* and *BRCA2* genes account for 20–25 % of inherited breast cancers and about 10 % of all breast cancer cases. Detection of BRCA mutation carriers can lead to therapeutic interventions such as mastectomy, oophorectomy, hormonal prevention therapy, improved screening, and targeted therapies such as PARP-inhibition. We estimate that African Americans and Hispanics are 4–5 times less likely to receive BRCA screening, despite having similar mutation frequencies as non-Jewish Caucasians, who have higher breast cancer mortality. To begin addressing this health disparity, we initiated a nationwide trial of BRCA testing of Latin American women with breast cancer. Patients were recruited through community organizations, clinics, public events, and by mail and Internet. Subjects completed the consent process and questionnaire, and provided a saliva sample by mail or in person. DNA from 120 subjects was used to sequence the entirety of *BRCA1* and *BRCA2* coding regions and splice sites, and validate pathogenic mutations, with a total material cost of $85/subject. Subjects ranged in age from 23 to 81 years (mean age, 51 years), 6 % had bilateral disease, 57 % were ER/PR+, 23 % HER2+, and 17 % had triple-negative disease.

**Results:**

A total of seven different predicted deleterious mutations were identified, one newly described and the rest rare. In addition, four variants of unknown effect were found.

**Conclusions:**

Application of this strategy on a larger scale could lead to improved cancer care of minority and underserved populations.

**Electronic supplementary material:**

The online version of this article (doi:10.1186/s13742-015-0088-z) contains supplementary material, which is available to authorized users.

## Background

Mutations in the *BRCA1* and *BRCA2* genes result in predisposition to breast and ovarian cancers [1, 2]. In addition, there is increasing evidence that BRCA mutations confer risk for cancers of the prostate, pancreas, stomach and skin [3–5]; there is also suggestive evidence for their involvement in esophageal and gastric cancers [6]. In Caucasian and Asian ethnicities, BRCA mutations are associated with basal-type and/or –triple-negative disease (in which the estrogen, progesterone, and HER2 receptors are absent from the tumor); however, the nature of this relationship in other ethnicities is understudied [7–10].

The identification of *BRCA1/2* carriers is a critical component of breast and ovarian cancer prevention as there are multiple screening, surgical, and chemoprevention strategies that can be employed. Magnetic resonance imaging (MRI) screening is effective in detecting early cancers in *BRCA1/2* carriers; mastectomy and oophorectomy can reduce ovarian cancers; and estrogen inhibition can reduce both type of malignancy [11]. However, relatively few minorities have participated in these prevention studies.

Although African American and Hispanic women have a lower incidence of breast cancer, they have a higher mortality. Triple-negative breast cancer is more common in African Americans and Hispanic women, accounting, in part, for this health disparity [12–16]. A study of over 45,000 women referred for *BRCA1* and *BRCA2* mutation screening from 2006 to 2008, found that 13–16 % of African Americans, Native Americans and Hispanics possess disease-causing mutations and a high rate of variants of unknown significance [17]. An independent study of 389 African Americans and 425 Hispanic women with incomplete gene sequencing, found that 8–10 % of high-risk subjects have a *BRCA1* or *BRCA2* gene mutation, and a small Puerto Rican study reported a 52 % mutation rate [18, 19]. Common recurrent mutations, including large deletions in *BRCA1* and *BRCA2*, exist in Hispanic/Latin American communities, which accounted for 35–45 % of the mutation carriers [17–20].

The BRCA1 and BRCA2 proteins play a role in the repair of double-stranded breaks in DNA. A synthetic lethal strategy for cancer therapy has been developed using DNA damaging chemotherapy agents to cause single-stranded breaks, combined with poly-ADP ribose polymerase (PARP) inhibitors, to inhibit single-stranded DNA repair. This approach may be particularly effective in BRCA mutation carriers, as the tumor will be unable to repair the double-stranded breaks [21, 22]. Clinical trials of PARP inhibitors demonstrate partial response or stable disease in breast, ovarian and prostate cancer subjects [23–27].

Mutations in *BRCA1* and *BRCA2* have been detected with a variety of techniques including multiple mutation scanning methods and Sanger sequencing (reviewed in [28]). Next-generation sequencing (NGS) has the benefit of high-throughput, automated sequence analysis, and single strand reads. DNA capture, droplet PCR and multiplex PCR methods of template preparation, and sequencing on 454, Illumina and Ion Torrent platforms have all been employed [29–32]. A clinical diagnostic laboratory validation of the Ion Torrent platform demonstrated an absence of false negatives and a 10 % false positive rate [33]. With the availability of a three-tube multiplex for the complete *BRCA1* and *BRCA2* genes we sought to apply this approach to a cohort of Hispanic/Latin American breast cancer patients.

## Data description

The data involve variants in the coding and flanking intron sequences of the human *BRCA1* and *BRCA2* genes in Hispanic subjects with breast cancer. The sequence was identified through amplification, library preparation and semi-conductor sequencing on an Ion Torrent Personal Genome Machine (PGM) Sequencer (Thermo Fisher Scientific) to an average (for all samples and amplicons of 293-307X) coverage in runs with 92 samples/chip and 466X with 46 samples/chip. Excluding samples giving fewer than 20,000 total reads, 1 amplicon of *BRCA1* was below 100x average reads (beginning of exon 2, containing 32 bp of the 5’UTR and 1 splice acceptor site); and 4 amplicons of *BRCA2* had <100× average, covering 261 bp of coding region and 5 splice sites. Therefore, the coverage of the coding regions is 100 % for *BRCA1* and 98 % for *BRCA2*.

The data consist of raw sequence reads mapped to the human genome, and the resulting BAM files. These files were used to predict sequence variants using the Torrent Suite Variant Caller (TSVC) and a modified Genome Analysis Tool Kit (GATK) variant caller, optimized for PGM data. For SNPs, the two variant callers (VCs) give virtually identical results, each calling one intronic SNP the other missed (both present on manual inspection). GATK is known to be ineffective at calling indels on the Ion Torrent platform. Parameter files for TSVC are given, as well as the raw and annotated variant files. Variants were manually examined in the Integrated Genome Viewer (IGV) and selected screen shots are provided. Rare variants were annotated to be Deleterious, Probably Benign, or Benign through inspection of appropriate databases (see Methods). Deleterious variants were validated by Sanger sequencing and displayed in Mutation Surveyor (SoftGenetics). Information on variants has been deposited in the LOVD ID #0000058963 [34].

Clinical data consists of information on the pathology of the tumor extracted from pathology reports, and results of a questionnaire administered by study personnel. Data are managed in a FileMaker relational database, and information on mutation carriers was double checked for accuracy. The composite information is displayed in Table 2, as well as the age-of-onset, pathology, hormone receptor status, and family history of cancer status of those subjects with mutations.

## Analyses

### Study design and patient population

**Fig. 1:**
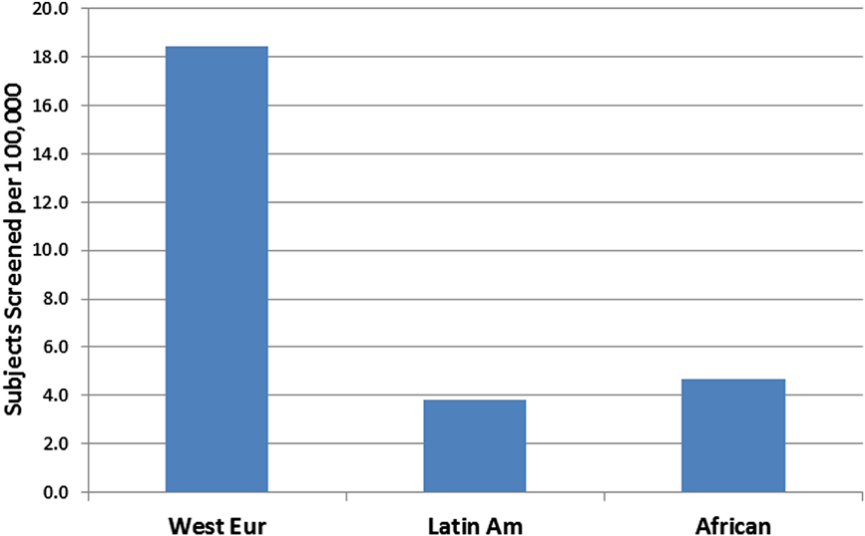
BRCA screening by ethnicity. The numbers of Western European (West Eur) women, Latin American (Latin Am), and African American (African) women who received BRCA screening per 100,000 population between 2006 and 2008, covered by Hall et al. [17] is displayed (see Table 1 for details)

To estimate the participation rate of minorities in *BRCA1* and *BRCA2* testing we used data from Hall et al. [35], on 64,717 non-Ashkenazi women receiving testing at Myriad Genetic Laboratories between the years 2006 and 2008, in order to calculate participation. Women of Western European descent made up 78 % of the subjects receiving testing during this time period. Latin American and African American women each made up only 4 % of the samples, despite representing 16 and 13 % of the US population, respectively. This represents 18.4 European Americans screened/100,000 as compared to 3.8/100,000 for Hispanics and 4.7/100,000 for African Americans (Table 1). Therefore, Hispanic and African American women are 4–5 times less likely to receive BRCA genetic testing than Western European women (Fig. 1). Economic factors, education, concern about genetic testing, and insurance coverage are likely to play roles in this deficit.

**Table 1:** BRCA screening by ethnicity

	West Eur	Cent Eur	Latin Am	African	Asian	Nat Am	Mid East	All
BRCA1	2501	336	185	180	75	44	30	3351
BRCA2	1899	214	105	100	75	35	16	2444
Total mutant	4400	550	290	280	150	79	46	5795
Subjects	36235	4066	1936	1767	1183	597	492	46276
% Mutation	12 %	14 %	15 %	16 %	13 %	13 %	9 %	13 %
Ratio BRCA1/2	1.32	1.57	1.76	1.80	1.00	1.26	1.88	1.37
% Sample	78 %	9 %	4 %	4 %	3 %	1 %	1 %	
US pop (1000s)	196817		50477	37686	14465	2247		
Screened/100,000	18.4		3.84	4.69	8.18			
Ratio West Eur/minority			4.80	3.93	2.25			

Data from Hall et al. [17] on BRCA screening and mutations identified by ethnic group were used to calculate the ratio of BRCA1/BRCA2 mutations (ratio BRCA1/2), and the percentage of the total sample represented by that ethnicity (% sample). The size of selected ethnic groups according to the US Census in thousands (US Pop (1000s)) divided by the number of subjects yields the number screened per 100,000 (Screened/100,000). The ratio of Western Europeans (West Eur) screened to Latin Americans (Latin Am), African Americans (African), and Asian American (Asians) was calculated by dividing Screened/100,000 for West Eur (18.4) by the corresponding figure of the minority population (Ratio West Eur/minority). Including the Central European (Cent Eur) women in with the West Eur women raises the minority ratios slightly (not shown)

We designed a clinical trial to address some of these issues, with recruitment of Latin American women through community organizations, clinics with large Hispanic populations, public events, and the Internet. Study materials were available in Spanish and English, and the patients were protected by a certificate of confidentiality [36]. We recruited a total of 135 subjects from 10 different states, and had an 88 % success rate in terms of completion and collection of consent forms, questionnaires, saliva samples, and pathology reports. The use of a saliva collection device that can be sent by regular mail allowed the materials cost of collection, shipping, and DNA extraction to be less than US$25.

The subjects had an age range at diagnosis of 23–81 years (mean 50.6 years); 60 % had a household income below $25,000; and 81 % were educated to high school level or less (Table 2). A total of 85 % of the subjects had invasive ductal carcinoma, 6 % bilateral disease, and 57 % ER/PR+, 23 % HER2+, and 17 % triple-negative disease. A family history of either a first or second-degree relative with breast cancer was identified in 35 % of cases.

**Table 2:** Demographics of subjects

	All	BRCA1 or 2 mutation	
		Yes	No
Patients	120	12 (10 %)	108 (90 %)
Triple-negative	20 (17 %)	6 (50 %)	14 (13 %)*
ER&PR positive	67 (57 %)	3 (25 %)	64 (61 %)*
HER2 positive	27 (23 %)	0	27 (26 %)*
Histology			
Invasive Ductal Carcinoma (IDC)	98 (85 %)	11 (92 %)	87 (82 %)
Ductal Carcinoma *In Situ* (DCIS)	11 (9 %)	0	11 (10 %)
Invasive Lobular Carcinoma (ILC)	4 (3 %)	0	4 (4 %)
Other/rare	4 (3 %)	1 (8 %)	3 (3 %)
Age at diagnosis	50.6 ± 11.6	43.7 ± 11.8	51.4 ± 11.3*
Age at first menses	12.8 ± 1.6	12.3 ± 1.4	12.8 ± 1.6
Education			
High school diploma or less	95 (81 %)	8 (66 %)	87 (82 %)
Beyond high school	23 (19 %)	4 (34 %)	19 (18 %)
Household Income			
< $25,000	61 (60 %)	1 (10 %)	60 (65 %)
> $25,000	41 (40 %)	9 (90 %)	32 (35 %)
Family history of BRCA	42 (35 %)	6 (50 %)	36 (34 %)

*Significantly different, *P* < 0.05

### DNA sequencing of BRCA1 and BRCA2

**Fig. 2:**
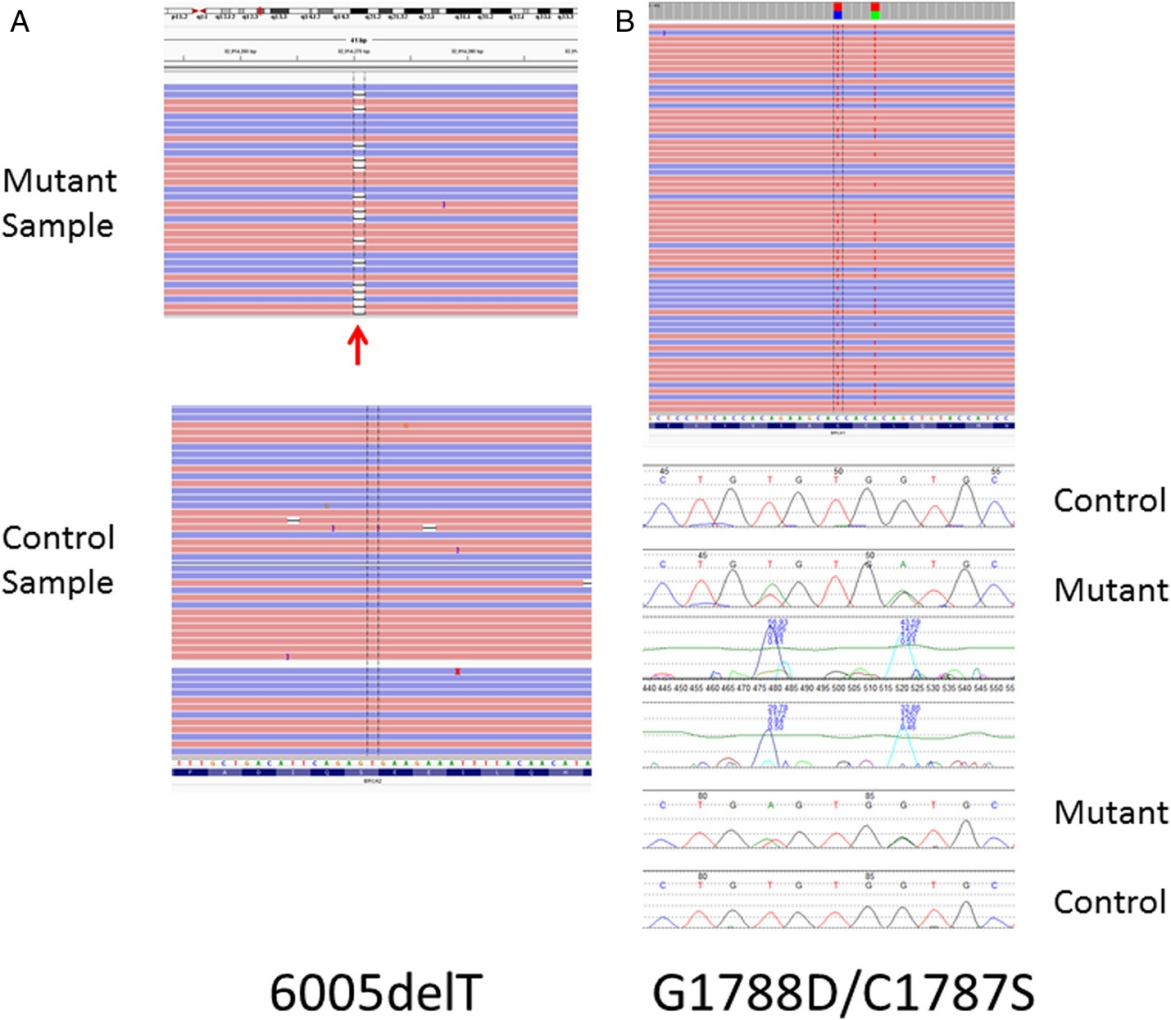
Selected *BRCA2* mutations. The Ion Torrent data displayed in IGV [51] is shown for the newly described 6005delT mutation in the left panel, **a**. The display shows individual forward (F) sequence reads in red and reverse (R) reads in blue. The 6005delT mutation can be seen as a gap in the sequence (arrow) in approximately half of the F and R reads – this is consistent with a heterozygous mutation. Sanger sequencing (not shown) confirmed this mutation. **b**. Both the Ion Torrent (above) and Sanger sequence (displayed in Mutation Explorer, SoftGenetics, below) for the C1787S and G1788D mutations are displayed in the right panel. The co-occurrence of the C1787S and G1788D variants on the same allele can be clearly seen in the Ion Torrent reads, whereas phase cannot be determined from the Sanger traces. Note: *BRCA1* is in reverse orientation in the genome, and so IGV display is of the reverse complement

An aliquot of each DNA sample was stripped of all identifiers in order to comply with the requirements of the protocol. A previously validated panel of primers (Ion AmpliSeg BRCA1 and BRCA2 Community Panel) was used to amplify all coding exons and splice sites (24,143 bp) with an average coverage of 313–466X and 100 % coverage (>100X average) of the *BRCA1* and 98 % of the *BRCA2* coding sequence. Variants were predicted using the Torrent Variant Caller; all predicted frameshift and premature stop codon alleles, and all other variants represented at less than 5 % in the 1000genomes database [37] and with quality scores greater than 40 were manually examined in IGV, and predicted deleterious variants were further confirmed by manual Sanger sequencing. A total of seven clearly deleterious alleles were identified, including a newly described allele: a single nucleotide deletion in *BRCA1* (6005delT, c.5777delT). The other six alleles were unique and, except for 189del11 in *BRCA1* and E1308X in *BRCA2*, all represent mutations uncommonly seen in these genes (Fig. 2, Table 3). Six of the seven mutations are frameshift or termination codons. The one missense variant is a compound allele C1787S and G1788D. These alleles have been reported five times in BIC [38] and have been proposed to be in cis on the same allele. NGS confirms this (Fig. 2).

**Fig. 3:**
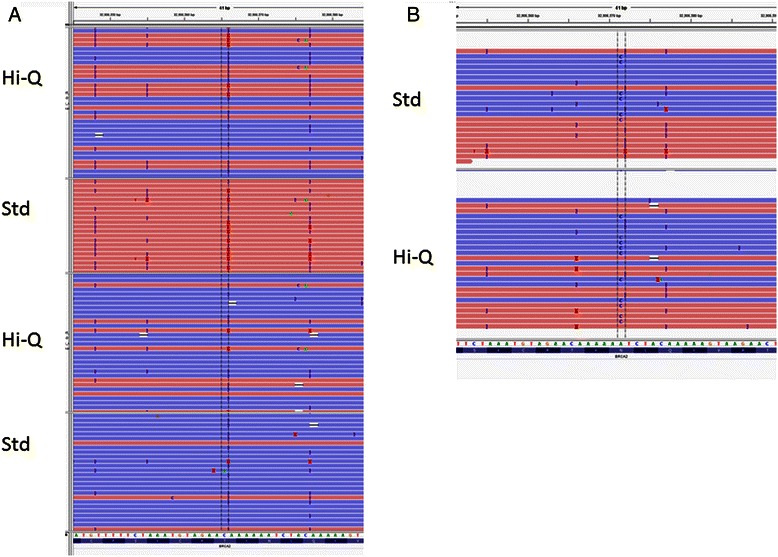
Screenshots of selected variants. **a**, A region with multiple homopolymers in *BRCA1* is shown, sequenced by the two enzyme formulations. **b**. A missense variant (benign) that was not called with the standard enzyme, but was by Hi-Q

**Table 3:** Classification of mutations and non-synonymous variants

Classification	Gene	Allele	IDS	HGVS cDNA	BIC	ClinVar	GVGD prediction	Path	TN	Bilat	Family Hist	Age diag
Pathogenic	BRCA1	188insAG	rs80357914	c.69_70insAG	2X			IDC&LC	No	No	No	35
Pathogenic	BRCA1	189del11	rs80359877	c.70_80del	11X			IDC	Yes	No	No	29
Pathogenic	BRCA1	5210delTG	rs80357710	c.5091_5092delTG	2X			IDC	Yes	No	1-2nd	32
Pathogenic	BRCA1	G1788D	rs80357069	c.5363G>A	5X			IDC	No	No	No	38
*	BRCA1	C1787S	rs80357065	c.5359T>A	5X							
Pathogenic	BRCA2	Q742X	rs80358494	c.2224C>T	2X			IDC		No	1-2nd	39
Pathogenic	BRCA2	E1308X	rs80358638	c.3922G>T	15X			IDC	No	No	No	41
Pathogenic	BRCA2	6005delT		c.5778delT	Not in BIC			IDC	No	No	No	35
VUS	BRCA1	E577Q		c.1729G>A	Not in BIC	No data	Class C0	IDC	Yes	No	1-2nd	49
VUS	BRCA2	F266L	rs587782433	c.796T>C	Not in BIC	1 VUS	Class C0	IDC	Yes	No	1-1st	61
VUS	BRCA2	D1781N	rs183478654	c.5341G>A	Not in BIC	1 VUS	Class C0	IDC&LC				
Likely Benign	BRCA1	L1844R	rs80357323	c.5531T>G	3X	2LB	Class C0					
Likely Benign/Benign	BRCA1	R504H	rs56272539	c.1511G>A	19X	2LB_1B	Class C0					
Likely Benign/Benign	BRCA1	T826K	rs28897683	c.2477C>A	38X	1 LB _3B	Class C0	IDC	No	No	4 1st, 2 2nd	66
Likely Benign/Benign	BRCA1	R841W	rs1800709	c.2521C>T	119X	1 LB_4B	Class C15					44
Likely Benign/Benign	BRCA1	I1275V	rs80357280	c.3823A>G	13X	1 LB_2B	Class C0	IDC	Yes	No	No	

All rare non-synonymous and coding region insertions and deletions were classified from a combined analysis of data from ClinVar, the Breast Cancer Information Core (BIC), and conservation. The subject’s pathology classification (Path), triple-negative status (TN), bilateral disease status (bilat), family history (Hist), and age of diagnosis (Age) are shown

*IDC* intraductal carcinoma, *LC* lobular carcinoma, *1* ^*st*^ first degree relative, *2* ^*nd*^ second degree relative

^a^C1787S/G1788D occur in cis in the same subject

^b^This patient also carries the *BRCA1* 189del11 mutation

Several rare missense alleles were found, but by using data in BIC and ClinVar, all but four could be excluded as: known; likely non-pathogenic variants; or those found in a sample with an existing mutation (Table 3). Two of these variants of unknown significance (VUS) are not present in BIC (*BRCA1* E577Q and *BRCA2* F266L). To further interrogate the variants of unknown significance, the Align GVGD site for evolutionary conservation [39] and LOVD [34] databases were examined. One of the VUS (*BRCA1* T826K) is listed as neutral in LOVD and the other three (F266L, E577Q and D1781N) are ranked as C0 (not conserved) in GVGD. It has been shown that an alignment of only primate sequences provides a potentially more appropriate model for human genetic variants [40]. Therefore, an alignment of all available primate BRCA1 and BRCA2 amino acid sequences was generated and the conservation of the residues determined. This analysis ranked all but one of the VUS (E577Q) as likely benign or benign (Table 3). E577Q is found in a patient with triple-negative disease and one second-degree relative with breast cancer.

Women with deleterious mutations were younger (43 versus 51 years of age, *P* = 0.029). Two of the seven patients with deleterious mutations had triple-negative disease (29 %); this is not significantly different from those patients without identified mutations. Only two of the patients with identified mutations had a family history of breast cancer, with one second-degree relative in each case. This frequency of a family history, 29 %, was not significantly different to that of the cohort as a whole.

### Testing of a highly accurate sequencing enzyme

Most sequencing technologies have a higher error rate at mononucleotide stretches of DNA. We previously documented this in a comparison of Ion Torrent, Illumina and Complete Genomics NGS machines [41]. While the latest version of TVC has eliminated most apparent 1 bp deletion artifacts, we identified a substantial number of apparent erroneous coding 1 bp insertions and deletions in mononucleotide regions, especially poly-A or poly-T repeats (Table 4).

**Table 4:** Hi-Q vs. Standard enzyme variant call comparison

Sample	CHR	Location	IDS	REF	VAR	Standard	QUAL	HiQ	QUAL	Context
Rare coding indels										
DL0099844	chr13	32906535			T	Artifact	10.3	No call		T5
DL0099806	chr13	32906547			T	Artifact	93.64	No call		T5
DL0099818	chr13	32906547			T	No call		Artifact	13	
DL0099791	chr13	32906565			A	Artifact	622.5	No call		A6
DL0099802	chr13	32906565			A	Artifact	737.8	No call		
DL0099818	chr13	32906565			A	Artifact	160.3	No call		
DL0099828	chr13	32906565			A	Artifact	793.8	No call		
DL0099833	chr13	32906565			A	Artifact	383.3	No call		
DL0099840	chr13	32906565			A	Artefact	744.6	No call		
DL0099874	chr13	32906565			A	Artefact	458.6	No call		
DL0099818	chr13	32906576			A	Artefact	158.9	No call		A5
DL0099879	chr13	32906576			A	Artefact	161.1	Artifact	293.2	
DL0099824	chr13	32906576			A	No call		Artifact	373.7	
DL0099842	chr13	32906576			A	No call		Artifact	212.6	
DL0099846	chr13	32906576			A	No call		Artifact	325.6	
DL0099851	chr13	32906576			A	No call		Artifact	288.0	
DL0099832	chr13	32906602			A	Artifact	101.0	No call		A7
DL0099854	chr13	32906602			A	Artifact	80.27	No call		
DL0099812	chr13	32906609			AAT	Artifact	11.8	No call		A7T4
DL0099867	chr13	32906609			AT	Artifact	19.39	No call		
DL0099873	chr13	32906609			AAT	Artifact	20.66	No call		
DL0099879	chr13	32906609			AT	Artifact	45.22	No call		
DL0099806	chr13	32906647			A	Artifact	32.02	No call		A5
DL0099841	chr13	32906647			A	Artifact	33.75	No call		
DL0099808	chr13	32913668			A	Artifact	11.78	No call		A4
**DL0099791**	**chr13**	**32914270**		**T**		**Valid**	**627**	**Valid**	**583.7**	**agTg**
DL0099847	chr13	32929161			A	Artifact	13.91	No call		A6
DL0099795	chr13	32929287			A	Artifact	22.35	No call		A4
DL0099802	chr13	32929287			A	Artifact	12.56	No call		
DL0099804	chr13	32929287			A	Artifact	14.27	No call		
DL0099816	chr13	32929287			A	Artifact	16.41	No call		
DL0099821	chr13	32929287			A	Artifact	10.65	No call		
DL0099831	chr13	32929287			A	Artifact	10.4	No call		
DL0099839	chr13	32929287			A	Artifact	15.97	No call		
DL0099844	chr13	32929287			A	Artifact	16.08	No call		
DL0099880	chr13	32929287			A	Artifact	20.42	No call		
**DL0099874**	**chr17**	**41215952**	**rs80357710**	**CA**		**Valid**	**893**	**Valid**	**794.1**	**CA4**
DL0099847	chr17	41256244			T	Artifact	13.14	No call		T4
**DL0099801**	**chr17**	**41276044**	**rs80359877**	**CAGATGGGACA**		**Valid**	**856**	**Valid**	**611.1**	
**DL0099855**	**chr17**	**41276044**			**CT**	**Valid**	**483**	**Valid**	**608.1**	
**Rare SNVs**										
**DL0099801**	**chr13**	**32906446**	**rs28897705**	**T**	**G**	**Valid**	**348**	**Valid**	**469.2**	
DL0099870	chr13	32906571	rs55939572	A	C	No call		Artifact	41.04	
**DL0099797**	**chr13**	**32912414**	**rs80358638**	**G**	**T**	**Valid**	**316**	**Valid**	**343.4**	
**DL0099879**	**chr13**	**32912553**	**cosmic:69844,esp,rs80358656**	**C**	**T**	**Valid**	**343**	**Valid**	**405.2**	
DL0099858	chr13	32972695	rs80358387	A	G	**Valid**	520.1	**Valid**	608.6	
DL0099877	chr17	41197756	rs80357323	A	C	**Valid**	522.5	**Valid**	675.3	
**DL0099816**	**chr17**	**41201181**	**rs80357069**	**C**	**T**	**Valid**	**512**	**Valid**	**799.2**	
**DL0099816**	**chr17**	**41201185**	**rs80357065**	**A**	**T**	**Valid**	**519**	**Valid**	**805.8**	
DL0099879	chr17	41222976	rs80356968	A	G	**Valid**	560.3	**Valid**	495.7	
DL0099858	chr17	41243948	rs56214134	C	A	**Valid**	758.5	**Valid**	698.1	
DL0099836	chr17	41245071	rs28897683	G	T	**Valid**	325.6	**Valid**	248.3	
DL0099801	chr17	41246037	rs56272539	C	T	**Valid**	835.4	**Valid**	998.0	
DL0099838	chr17	41267755		T	A	**Valid**	731.1	**Valid**	910.6	

A set of 91 samples were amplified and the resulting library run on either the standard sequencing enzyme or Hi-Q. Variants were called by TVC4.0 with recommended settings optimized for Hi-Q enzyme (see Methods). Lines in bold were manually validated

*REF* reference base, *VAR* variant base. *QUAL* quality score of variant

A recently developed enzyme, Ion Hi-Q Sequencing Chemistry, has been designed by Life Technologies for higher accuracy with respect to insertions and deletions (indels) and homopolymers (Ion Hi-Q Sequencing Chemistry Technology Access Program Information). We sequenced the same library of 91 DNA samples using both the standard and the Hi-Q enzyme, and analyzed the results according to the manufacturer’s instructions. With the Hi-Q enzyme the number of false positives was lower especially for 1 bp deletion alleles (Table 4, Fig. 3). The use of Hi-Q could greatly streamline variant prediction by reducing the number of variants requiring manual review.

## Discussion

Rates of mortality, triple-negative disease, and *BRCA1* and *BRCA2* mutational and allelic diversity are all higher in African American and Hispanic populations. Although these populations could benefit significantly from genetic testing and screening, the combination of lower average income; lower insurance coverage; reduced knowledge of the benefits of testing; and mistrust of medical and government agencies, have led to a large disparity in participation [42–44]. From 64,717 women in the Myriad database, this under-participation is 3.9–4.8 times lower in these two populations, which together account for 29 % of the US population [35]. A similarly low rate of participation from the first 10,000 women tested at Myriad was noted by Forman and Hall [43].

To begin to address these issues in Hispanic populations, we designed a study with a number of potential advantages:

- a fully de-linked sample not requiring extensive counseling regarding BRCA testing;
- collection of saliva that can be completed in the home, community clinics or public events;
- bilingual study materials; and
- full protection of confidentiality.

Our recruitment success was very modest from online publicity (Facebook, clinicaltrials.gov), and few minority patients participate in public fund-raising events, such as Avon fundraising walks. However, partnerships with community groups such as Nueva Vida (Baltimore, Richmond) and the Latino Community Development Agency (Oklahoma City) were successful in recruiting a number of Hispanic women of diverse backgrounds. The remainder of the population samples were collected at the Texas Tech University Health Science Hospitals in Lubbock and El Paso, Texas. By sending and receiving saliva kits through the US mail we were able to keep the cost of the collection/shipping and DNA preparation materials to under $20 per subject. Targeted sequencing, performed in batch sizes of 90 samples, has a materials cost of approximately $50, making the total reagent cost under $100, including Sanger validation but excluding labor. This is an important factor if sample sizes in the order of thousands of subjects are to be eventually attained.

We did identify amplicons in *BRCA2* that performed poorly in most samples, having <100X average coverage, comprising 261 bp of coding region and 4 splice sites, and 1 splice site of *BRCA1* poorly covered. The initial run of 92 samples had three that underperformed and required repetition (3 %); however, these samples performed well on a second run. Our second run of 46 samples allowed the average coverage to increase from 313–361X to 466X. Running 46 samples on a single chip would raise the cost/sample by $5, and may be advisable for clinical testing.

The *BRCA1* gene contains multiple mononucleotide repeats, for which it is challenging to accurately detect 1 bp indels; these regions are the most problematic to detect for all current methods [36, 40, 41]. Early versions of the TVC demonstrated relatively higher error rates on homopolymer sequences [40, 41]. This performance has improved with subsequent version of TVC and three studies demonstrate that 70-130X coverage across homopolymer regions is sufficient to get accurate mutation calls in the *BRCA1* and *BRCA2* genes (Table 5, Additional file 1) [42, 43]. Using the 70X standard of Dacheva et al., besides the poor performing exons mentioned above, there are only 2 A5 homopolymer repeats in coding regions (*BRCA2*, exon 12, 65X average coverage) that are below this threshold in our data. In a small sample set, we show here that the Hi-Q formulation and current TVC analysis settings result in a substantial reduction in 1 bp indel calls, especially in false positive mononucleotide regions. Manual analysis of BAM files in IGV allows most of these variants to be excluded from consideration, although the difference in background between the two enzymes visible in IGV displays are not dramatic. The addition of overlapping amplicons in the most difficult regions could improve this result. Although we chose an amplicon-based method on Ion Torrent, the samples could also be run on multiple platforms or used in a capture-based method [29]. Color Genomics (https://getcolor.com/) now offers a clinical test of 19 breast cancer related genes for $249.

While this initial phase of the project was designed to maximize participation and diversity at a modest cost, it has the disadvantage that subjects with mutations could not be retrospectively identified in order to benefit from the testing. A second phase, in which identifiers will be retained and patients counseled about testing, will now be initiated. We expect recruitment to increase as several individuals and groups declined to participate in the unlinked arm of the study. Population growth; advancement in age of the US Hispanic populations; and large family size make it imperative that innovative means be employed to increase participation in clinical and genetic studies. The increasingly recognized involvement of germline mutations in *BRCA1* and *BRCA2* in diverse cancers, as well as the active design of targeted therapies, further adds to the need to recruit minority subjects.

To date there have been no large nationwide surveys of Latin American with breast cancer. In the report by Hall et al., only the identities of mutations reaching 4 % or greater were made available [35]. The Clinical Cancer Genetics Community Research Network collected samples and data from 746 patients from 14 clinics concentrated in Southwestern USA, and individual data from centers in Texas, California, and Puerto Rico [46]. Our study adds to the diversity of Hispanic BRCA mutations, and – interestingly – we did not find the most common allele in all mainland US Hispanic studies to date, the *BRCA1* 185delAG mutation. Of the six mutations we identified, only one, *BRCA2* E1308X, has been reported in multiple studies. A much larger study, incorporating all the regional and ethnic diversities of Hispanic populations, will have to be carried out to fully understand mutational diversity, and to aid in the classification of VUS.

Clearly, a comprehensive characterization of our samples will require copy number analysis to identify large insertion/deletion mutations. Recurrent large deletions in *BRCA1* have been found in both Mexican and Puerto Rican breast cancer patients [19, 20]. Rare germline mutations in other genes have been identified in familial breast cancer, and our samples could be used to scan these genes [32] or complete exomes or genomes.

While we and others have documented that the major next-generation sequencers do an excellent job in identifying single nucleotide variants, they can be deficient in the prediction of insertion and deletion variants, especially in mononucleotide repeats [41]. The *BRCA1* and *BRCA2* genes have numerous mononucleotide repeat regions, and these areas are rich in known mutations. Thus, a method to increase accuracy of sequencing in repeats would be welcome. We documented that the Hi-Q enzyme can achieve a significantly higher accuracy in sequencing through mononucleotide repeats. When combined with methods or specific assays for the most prevalent large deletions, a high percentage of germline mutations can be identified. A recent study with the same BRCA1/2 panel was tested in a diagnostics laboratory with high accuracy [47].

**Table 5:** Published studies using Ion Torrent sequencing on BRCA1 and BRCA2 indels

Publication	Ref.	known mutations	indels	1bp indels	Homopol. indels	Comments/Conclusions
Costa et al.	[33]	9	9	2	1	Largest indel is a 3bp homopolymer
Tarabeau et al.	[57]	48	35	25	22	9 in BRCA1, 13 in BRCA2, established 130X as minimum coverage to detect all variants
Dacheva et al.	[58]	7	10	5	4	Established minimum coverage of 70X to detect all variants
Kluska et al.	[59]	20	15	7	3	8 in BRCA1 (3 1bp) and 7 in BRCA2 (4 1bp)
Yeo et al.	[60]	3	3	2	0	Used multiple mappers and variant callers to show that high sensitivity and specificity can be obtained in BRCA1/2 with Ion Torrent sequencing, but no mutations are in homopolymers
Trujillano et al.	[47]	19	8	6	3	115 known validation samples and 95 unknown samples
Chan et al.	[61]		3	2	1	Compares Solid to PGM, estimates reagent cost as $123 (Solid) and $220 (PGM)
Bosdet et al.	[62]	517	18	5	4	Known variants includes SNPs. Establish 100X minimum coverage to find all variants
	Total		101	54	38	

All known publications using Ion Torrent sequencing instruments to sequence known BRCA1 and BRCA2 mutations are displayed. The number of insertion/deletion variants (indels), those of 1 base pair (1bp) and those in homopolymer regions (homopol.) are tabulated

In summary, we have succeeded in recruiting and anonymously testing diverse groups of Latin American women with breast cancer in the US, all at a materials cost of less than $100 for samples collection, shipment and sequencing, and confirmation. Using DNA sequencing, we found almost exclusively rare mutations, most of which have been observed in other studies. Expansion of this approach could be a component of a larger effort to improve the application of the benefits of genetic testing to Hispanic American women.

## Potential implications

The benefits of *BRCA1* and *BRCA2* testing for women with breast and/or ovarian cancers, women with a family history and/or elevated risk, or even all women, are evident [48]. A range of options for women at risk is currently available, including increased and more effective screening, risk reduction through hormone reduction therapy, and surgical intervention. As cancers in *BRCA1* and *BRCA2* mutation carrier subjects occur at an earlier age, identification, education and implementation of risk reduction has a high cost-to-benefit ratio in favor of benefit. By reducing the cost of testing, simplifying sample collection, and working with organizations and clinics focusing on Hispanic communities, we address some of the barriers to utilizing this technology. Extending this approach to larger populations, employing counseling and analysis in approved clinical genetics laboratories, could contribute to reducing the higher mortality from these cancers in minority populations.

## Methods

### Study design and patients

Hispanic patients with breast cancer were recruited through community organizations, dedicated clinics, public events (Avon Walk) and through online contacts. The study was approved by the National Cancer Institute (NCI) Institutional Review Board (IRB) as well as the IRBs of Texas Tech Health Science Center Hospitals at Lubbock and El Paso, and included a confidentiality agreement from the National Institutes for Health (NIH) [36]. Subjects were newly diagnosed or previously treated, and gave consent in both English and Spanish, with the protocol and questionnaire available in both languages. A validated questionnaire was used to capture data on reproductive health, education, income, and family history, and a pathology report was collected to capture data on pathology and estrogen, progesterone receptor status, as well as HER2 status.

### DNA collection and extraction

Saliva (~5 ml) was collected in Oragene collection devices (DNA-Genotek, Ontario, Canada), stored at room temperature, and shipped at ambient temperature through the US mail. A 0.5 ml aliquot was extracted according to manufacturer’s instructions and quantified by a NanoDrop (ND-1000) spectrophotometer (Thermo-Fisher, Wilmington, DE). Per the IRB protocol, DNA samples and selected clinical data were given new numbers unlinked to patient identifiers.

### DNA sequencing

Starting with 30 ng of genomic DNA, samples were processed according to the standard protocol for Ampliseq target amplification and library preparation using the targeted, multiplex Ion AmpliSeq BRCA1 and BRCA2 Community Panel. The panel contains 167 amplicons, covers 16.3 kb and provides 98–100 % coverage of the coding regions of the *BRCA1* and *BRCA2* genes [49]. The libraries were prepared following the manufacturer’s Ion AmpliSeq Library Preparation protocol (Life Technologies, Carlsbad, CA, USA) and individual samples were barcoded, pooled together for the template emulsion preparation, and then sequenced on a P1 chip and Ion Torrent PGM Sequencer (Thermo Fisher Scientific). Each run produced over 10 Gb of sequence data, and each sample had an average depth of coverage surpassing 500X. Raw sequencing reads generated by the Ion Torrent sequencer were quality and adapter-trimmed by Ion Torrent Suite, then aligned to the hg19 reference sequence by TMAP [50] using default parameters (parameter file provided). Resulting BAM files were merged according to sample names and processed through an in-house quality control (QC) and coverage analysis pipeline, which generated coverage summary plots and per sample per amplicon read count heatmaps (heatmaps provided). Aligned BAM files were left-aligned using the GATK LeftAlignIndels module. Amplicon primers were trimmed from aligned reads. Variant calls and filtering was made by Torrent Variant Caller 4.0 (TSVC). Two slightly difference parameter settings were used for standard sequencing enzyme and Hi-Q enzyme. In the Hi-Q enzyme parameter set, variant recalibration was enabled. All other parameters, such as minimum coverage, minimum alternative allele frequency, and strand bias were the same between the two settings (parameter files provided). Filtered variants were annotated by the Glu Genetics annotation pipeline [51].

### Sequence analysis

Predicted variants were manually reviewed in IGV [51] and manually confirmed variants examined for data in the Breast Cancer Information Core (BIC) [38], and the ClinVar database [52]. Further analysis of variants was performed using the Leiden Open Variation Database (LOVD) and ALIGN-GVGD [34, 39, 54, 55]. Selected sites for common mutations were manually examined across all samples to ensure that the false positive rate was low, and no additional variants detected. Selected mutations were repeated by Sanger sequencing and gave identical results to the PGM sequence. The newly described mutation, BRCA2 6005delT has been submitted to LOVD (Variant ID 0000058963).

### Data analysis

Data was compiled in a relational database (Filemaker) and statistical analysis performed in STATA (StataCorp LP, College Station, TX, USA).

## Availability of supporting data

Supporting data including BAM files of standard and Hi-Q enzyme libraries and ABI Trace files of validated mutations are available from the *GigaScience* GigaDB [56]. The newly described BRCA2 6005delT mutation has been submitted to LOVD (Variant ID 0000058963).

## Additional file

Additional file 1: Table S1. Mutation details for variants in Table 5. (DOCX 27 kb)

## Abbreviations

BIC: Breast Information Core
ER: Estogen receptor
PR: Progesterone receptor
GATK: Genome Analysis Tool Kit
IGV: Integrated Genome Viewer
IRB: Institutional Review Board
LOVD: Leiden Open Variation Database
MRI: Magnetic resonance imaging
NCI: National Cancer Institute
NGS: Next-generation sequencing
NIH: National Institutes for Health
PARP: poly-ADP ribose polymerase
PGM: Personal Genome Machine
TSVC: Torrent Suite Variant Caller
VC: Variant caller
VUS: Variants of unknown significance
