# Supplementary material for: Addressing health disparities in Hispanic breast cancer: accurate and inexpensive sequencing of BRCA1 and BRCA2
Source: Gigascience. 2015 Nov 4;4:50. doi: 10.1186/s13742-015-0088-z (PMC4634732; doi:10.1186/s13742-015-0088-z)
Supplement: Additional file 1: Table S1. — Mutation details for variants in Table 5. (DOCX 27 kb) [file 13742_2015_88_MOESM1_ESM.docx]

Table S1. Mutation details for variants in Table 5.

| **Reference** | **Gene** | **Mutation** | **Size** | **Homopolymer** | **Location** | **dbSNPID** |
| --- | --- | --- | --- | --- | --- | --- |
| Yeo et al. | BRCA2 | c.3846_3847del | 2 | T2 | Exon 11 |  |
|  | BRCA2 | *c.7696_7697insA* | 1 | N | Exon 16 |  |
|  | BRCA1 | *c.3424delG* | 1 | N | Exon 11 |  |
|  |  |  |  |  |  |  |
| Costa et al. | BRCA1 | c.470_471delCT p.S157X | 2 | (CT)3 |  |  |
|  | BRCA1 | c.2037delinsCC p.K679NfsX3 | 2 | N | Exon 11 |  |
|  | BRCA1 | c.5030_5033delCAAT p.T1677IfsX1 | 4 | N |  |  |
|  | BRCA1 | c.5266_5267insC p.Q1756PfsX1829 | 1 | C3 |  |  |
|  | BRCA2 | c.156–157insAlu p.H52fsX137 | Alu insertion | N |  |  |
|  | BRCA2 | c.658_659delGT p.V220IfsX223 | 2 | N |  |  |
|  | BRCA2 | c.2808_2811delACAA p.K936fsX958 | 4 | N |  |  |
|  | BRCA2 | c.4380_4381delTT p.F1460fs4 | 2 | N |  |  |
|  | BRCA2 | c.7631delG p.G2544AfsX2550 | 1 | G2 | Exon 16 |  |
|  |  |  |  |  |  |  |
| Tarabeaux | BRCA1 | c.19_47del | 28 | N |  |  |
|  | BRCA1 | c.68_69del | 2 | N |  |  |
|  | BRCA1 | c.1121del | 1 | N |  |  |
|  | BRCA1 | c.3013del | 1 | N |  |  |
|  | BRCA1 | c.3416_3427delinsC | 11 | N |  |  |
|  | BRCA1 | c.3481_3491del | 11 | N |  |  |
|  | BRCA1 | c.3680_3729dup | 49 | N |  |  |
|  | BRCA1 | c.3839_3843delinsAGGC | 4 | N |  |  |
|  | BRCA1 | c.4243_4281dup | 38 | N |  |  |
|  | BRCA1 | c.5030_5033del | 3 | N |  |  |
|  | BRCA1 | c.5266dupC | 1 | C3 |  | [rs397507246](http://www.ncbi.nlm.nih.gov/projects/SNP/snp_ref.cgi?rs=397507246) |
|  | BRCA1 | c.1016dupA | 1 | A7 | Exon 11 |  |
|  | BRCA1 | c.1961dupA | 1 | A8 | Exon 11 |  |
|  | BRCA1 | c.1961delA | 1 | A8 | Exon 11 |  |
|  | BRCA1 | c.2071delA | 1 | A4 | Exon 11 | rs80357688 |
|  | BRCA1 | c.211del | 1 | A4 | Exon 5 |  |
|  | BRCA1 | c.3926delC | 1 | C2 | Exon 11 | rs80357815 |
|  | BRCA1 | c.2429delC | 1 | C4 | Exon 11 |  |
|  | BRCA1 | c.3285delA | 1 | A3 | Exon 11 |  |
|  | BRCA2 | c.3645_3646delinsTAAAAAG | 7 | N |  |  |
|  | BRCA2 | c.5835_5842dup | 7 | N |  |  |
|  | BRCA2 | c.161delA | 1 | A5 | Exon 3 |  |
|  | BRCA2 | c.994dupA | 1 | A7 | Exon 10 |  |
|  | BRCA2 | c.1231delA | 1 | A4 | Exon 10 |  |
|  | BRCA2 | c.1593dupA | 1 | A6 | Exon 10 |  |
|  | BRCA2 | c.1813dupA | 1 | A8 | Exon 10 | rs80359308 |
|  | BRCA2 | c.1929delG | 1 | N | Exon 11 | rs80359316 |
|  | BRCA2 | c.2175dupA | 1 | A6 | Exon 11 | [rs276174819](http://www.ncbi.nlm.nih.gov/projects/SNP/snp_ref.cgi?rs=276174819) |
|  | BRCA2 | c.2588dupA | 1 | A7 | Exon 11 |  |
|  | BRCA2 | c.4284dupT | 1 | T6 | Exon 11 | rs80359440 |
|  | BRCA2 | c.5351delA | 1 | A6 | Exon 11 | rs80359509 |
|  | BRCA2 | c.5351dupA | 1 | A6 | Exon 11 |  |
|  | BRCA2 | c.6373dupA | 1 | A6 | Exon 11 | rs80359577 |
|  | BRCA2 | c.7680dupT | 1 | T2 | Exon 16 |  |
|  | BRCA2 | c.8207delC | 1 | C4 | Exon 18 |  |
|  |  |  |  |  |  |  |
| **Dacheva** | BRCA1 | c.5263_5264insC | 1 | C3 | Exon 20 | rs80357906 |
|  | BRCA1 | c.548-58delT | 1 | T6 | Intron 8 | rs8176144 |
|  | BRCA1 | c.465_466delA | 1 | A2 | Exon 8 |  |
|  | BRCA2 | c.5722_5723delCT | 2 | N | Exon 11 | rs80359531 |
|  | BRCA2 | c.5851_5854delAGTT | 4 | N | Exon 11 | rs80359544 |
|  | BRCA2 | c.6841+80_6841+83delTTAA | 4 | N | Intron 11 | rs11571661 |
|  | BRCA2 | c.7007+134_7007+135insTTATAAAAT | 9 | N | Intron 13 | rs11571680 |
|  | BRCA2 | c.7913_7917delTTCCT | 5 | N | Exon 17 | rs80359687 |
|  | BRCA2 | c.8632+148delT | 1 |  | Intron 20 |  |
|  | BRCA2 | c.9682delA | 1 | A3 | Exon 27 | rs398122618 |
|  |  |  |  |  |  |  |
| **Bosdet** | BRCA1 | c.68_69delAG | 2 | N |  |  |
|  | BRCA1 | c.927_929delACAinsAC | 1 | N |  |  |
|  | BRCA1 | c.1018delG | 1 | G2 |  | [rs80357774](http://www.ncbi.nlm.nih.gov/projects/SNP/snp_ref.cgi?rs=80357774) |
|  | BRCA1 | c.2241delC | 1 | C4 |  | [rs80357650](http://www.ncbi.nlm.nih.gov/projects/SNP/snp_ref.cgi?rs=80357650) |
|  | BRCA1 | c.2411_2412delAG | 2 | N |  |  |
|  | BRCA1 | c.3648dupA | 1 | N |  |  |
|  | BRCA1 | c.3770_3771delAG | 2 | N |  |  |
|  | BRCA1 | c.3856_3859delAGTG | 4 | N |  |  |
|  | BRCA1 | c.5266dupC | 1 | C3 |  | rs397507246 |
|  | BRCA2 | c.574_575delAT | 2 | N |  |  |
|  | BRCA2 | c.2808_2811delACAA | 4 | N |  |  |
|  | BRCA2 | c.3847_3848delGT | 2 | N |  |  |
|  | BRCA2 | c.4146_4148delAGA | 3 | N |  |  |
|  | BRCA2 | c.4478_4481delAAAG | 4 | N |  |  |
|  | BRCA2 | c.5350_5351delAA | 2 | A7 |  | rs80359507 |
|  | BRCA2 | c.5576_5580delTTAAAinsA | 4 | N |  |  |
|  | BRCA2 | c.6082_6086delGAAGA | 5 | N |  |  |
|  | BRCA2 | c.7069_7070delCT | 2 | N |  |  |
|  |  |  |  |  |  |  |
| **Kluska** | BRCA1 | c.66_67delAG | 2 | N |  |  |
|  | BRCA1 | c.3700_3704del5 | 5 | N |  |  |
|  | BRCA1 | c.3756delGTCT | 4 | N |  |  |
|  | BRCA1 | c.3777delT | 1 | T3 |  | rs80357798 |
|  | BRCA1 | c.4035delA | 1 | A2 |  | rs80357711 |
|  | BRCA1 | c.4041delAG | 2 | N |  |  |
|  | BRCA1 | c.4065delTCAA | 4 | N |  |  |
|  | BRCA1 | c.5263delC | 1 | C3 | Exon 20 |  |
|  | BRCA2 | c.5239insT | 1 | N | Exon 11 | rs80359500 |
|  | BRCA2 | c.5946delT | 1 | N | Exon 11 | rs80359550 |
|  | BRCA2 | c.5964delAT | 2 | N |  |  |
|  | BRCA2 | c.6447delTA | 2 | N |  |  |
|  | BRCA2 | c.7910del5 | 5 | N |  |  |
|  | BRCA2 | c.8924delT | 1 | N | Exon 22 |  |
|  | BRCA2 | c.9402delT | 1 | N | Exon 25 |  |
|  |  |  |  |  |  |  |
| **Trujiano** | BRCA1 | c.2071del | 1 | A4 | Exon 10 | rs80357688 |
|  | BRCA1 | c.4236del | 1 | N |  |  |
|  | BRCA1 | c.5177_5180del | 4 | N |  | rs80357975 |
|  | BRCA1 | c.5266dup | 1 | C3 |  | rs80357906 |
|  | BRCA2 | c.467_468insT | 1 | N | Exon 5 |  |
|  | BRCA2 | c.965_968del | 4 | N | Exon 10 |  |
|  | BRCA2 | c.7068_7069del | 1 | T3 | Exon 14 |  |
|  | BRCA2 | c.9097_9098insT | 1 | N | Ex. 23 |  |
|  |  |  |  |  |  |  |
| **Chan** | BRCA1 | 3543delG | 1 | G2 | Exon 11 |  |
|  | BRCA2 | 4073del2 | 2 | N |  |  |
|  | BRCA2 | 7925insA | 1 | N |  |  |
